# Validation of orthopaedic surgeons’ assessment of cognitive function in patients with acute hip fracture

**DOI:** 10.1186/s12891-019-2633-x

**Published:** 2019-06-01

**Authors:** Målfrid Holen Kristoffersen, Eva Dybvik, Ole Martin Steihaug, Christoffer Andreas Bartz-Johannesen, Mette Irene Martinsen, Anette Hylen Ranhoff, Lars Birger Engesæter, Jan-Erik Gjertsen

**Affiliations:** 1 Norwegian Hip Fracture Register, Department of Orthopaedic Surgery, Haukeland University Hospital, Jonas Lies vei 65, N 5021 Bergen, Norway; 2 Department of Clinical Medicine, Faculty of Medicine, University of Bergen, Haukelandsveien 28, N 5009 Bergen, Norway; 3 Haraldsplass Deaconess Hospital, Ulriksdal 8, N 5009 Bergen, Norway; 4 Diakonhjemmet Hospital, Postboks 23 Vindern, N 0319 Oslo, Norway; 5 Department of Clinical Sciences, Faculty of Medicine, University of Bergen, Haukelandsveien 28, N 5009 Bergen, Norway

**Keywords:** Hip fracture, Orthopaedic surgeon, Mental status, Dementia tests

## Abstract

**Background:**

About one fourth of patients with hip fracture have cognitive impairment. These patients are at higher risk of surgical and medical complications and are often excluded from participating in clinical research. The aim of the present study was to investigate orthopaedic surgeons’ ability to determine the cognitive status of patients with acute hip fracture and to compare the treatment given to patients with and without cognitive impairment.

**Methods:**

The cognitive function of 1474 hip fracture patients reported by the orthopaedic surgeons to the nationwide Norwegian Hip Fracture Register was compared with data registered in quality databases in two hospitals with orthogeriatric service on the same patients. Cognitive function registered in the quality databases was determined either by the short form of the Informant Questionnaire on Cognitive Decline in the Elderly (IQCODE) or by pre-fracture diagnosis of dementia. The information registered in the quality databases was defined as the reference standard. Cognitive function in the Norwegian Hip Fracture Register was reported as: Chronic cognitive impairment? “Yes”, “Uncertain” or “No” by the orthopaedic surgeons. Sensitivity, specificity, negative and positive predictive values for chronic cognitive impairment reported to the Norwegian Hip Fracture Register by the orthopaedic surgeons was calculated.

Baseline data and treatment of hip fractures in patients with and without cognitive impairment in the Norwegian Hip Fracture Register were compared.

**Results:**

Orthopaedic surgeons reported chronic cognitive impairment in 31% of the patients.

Using documented dementia or IQCODE > 4.0 as the reference, this assessment of cognitive impairment by the orthopaedic surgeons had a sensitivity of 69%, a specificity of 90%, a positive predictive value of 78%, and a negative predictive value of 84% compared to information registered in the two hospital quality databases.

There were no differences in type of hip fracture or type of surgical treatment by cognitive function.

**Conclusion:**

The treatment of hip fractures was similar in patients with chronic cognitive impairment and cognitively well-functioning patients. The surgeons had an acceptable ability to identify and report chronic cognitive impairment in the peri-operative period, indicating that the Norwegian Hip Fracture Register is a valuable resource for future registry-based research also on hip fracture patients with chronic cognitive impairment.

## Background

Norway, with 5.3 million inhabitants, has one of the highest incidences of hip fractures in the world [1]. Annually, about 9000 patients sustain a hip fracture in Norway with an average age of 80 years and less than 40% of these patients were classified to be in the healthiest groups (ASA 1 and 2) [2]. Studies have reported that 19–37% of hip fracture patients have cognitive impairment [3, 4]. Cognitive impairment is a known risk factor for sustaining a hip fracture [5–7]. Previous studies have reported lower quality of life after hip fracture in patients with cognitive impairment compared to cognitively well-functioning patients [8–10].

With an ageing population, there will also be an increase in the proportion of people with cognitive impairment [11]. Still, patients with cognitive impairment and dementia are excluded from 8 of 10 hip fracture studies [7]. One reason may be the difficulty of evaluating the patients’ cognitive function in the peri-operative period. Cognitive impairment is a term used for both acute and chronic impairment in cognitive function. Delirium is an acute state of confusion that frequently occurs during hospitalization for hip fracture and which makes it challenging to determine the patients` habitual cognitive function [12]. Nordic studies have reported an overall incidence of delirium of 21–50% in hip fracture patients [12, 13]. Bitsch et al. reported an overall incidence of delirium of 36% in hip fracture patients [14]. A diagnosis of dementia requires a cognitive impairment of more than 6 months duration and of sufficient severity to interfere with activities of daily living. Patients with a hip fracture are at risk of developing dementia postoperatively and delirium can play an important role in this development [15, 16]. A study on hip fracture patients without pre-fracture cognitive impairment reported that 38% of the patients that developed delirium during hospitalization were diagnosed with dementia 6 months later [16]. Hip fracture patients with cognitive impairment have higher risk of both surgical complications such as surgical site infections, and non-surgical complications such as respiratory complications [11], as well as delirium [12]. Further, patients with delirium have increased risk of post-operative complications such as infection, dislocation of hip prostheses and new fractures due to falls [17]. Both patients with dementia and delirium therefore need extra attention during their hospital stay and it is important that surgeons and other health professionals are able to identify these patients early to optimize care and try to minimize risk for complications [12, 18, 19].

The Norwegian Hip Fracture Register (NHFR) has registered hip fractures on a national basis since 2005 [20], and cognitive function is reported to the registry by the surgeon after each operation for a hip fracture. Our aim was to investigate the surgeons’ ability to determine cognitive function in the peri-operative period in patients with acute hip fractures. We compared chronic cognitive function reported by the surgeons to the NHFR with data on chronic cognitive function assessed by special trained nurses and geriatricians and registered in two local hospital quality improvement databases as the reference standard for the same patients.

Our aim in the present study was to investigate orthopaedic surgeons’ ability to determine cognitive function in patients with an acute hip fracture, and thereby also to validate the information on cognitive function reported to the NHFR.

## Methods

### Data from hospital quality databases

Data from two hospital quality databases for hip fracture patients, Haraldsplass Deaconess Hospital (HDH) in Bergen, Norway and Diakonhjemmet Hospital (DH) in Oslo, Norway were used as the reference standard for the patients` cognitive function. Both hospitals had orthogeriatric units, staffed by orthopaedic surgeons and geriatric consultants. The databases contain data such as date of operation, comorbidity, chronic cognitive impairment, medical complications and length of stay. The databases are managed by special trained nurses in cooperation with geriatricians and information is registered during the patients’ hospital stay. The patients’ pre-fracture cognitive function was assessed by short form of the Informant Questionnaire on Cognitive Decline in the Elderly (IQCODE) [21].

The IQCODE is an instrument containing 16 questions about change in everyday tasks related to cognitive ability compared to 10 years previously [22, 23]. The form is filled in by a close relative. Each question is scored from 1 to 5 with values less than 3 indicating better cognitive performance, while a score of 3 indicates similar performance and values greater than 3 indicate cognitive impairment. The form containing IQCODE was usually collected postoperatively by the non-surgical staff of the orthogeriatric ward. Gold standard evaluation of cognitive impairment requires a detailed history and assessment by trained health care personnel. IQCODE is a validated assessment tool that can give an indication of cognitive impairment prior to the hip fracture when the patient was in her/his habitual state. However, IQCODE on its own is not sufficient to diagnose dementia [21].

At DH, the quality database in addition to the IQCODE contained information on dementia diagnosis (Dementia: Yes or No) obtained from the patients` medical charts. Consequently, at this hospital some patients with information on advanced dementia in the medical chart were not assessed using the IQCODE.

Peri-operatively collected data on cognitive impairment in the quality databases were considered the reference standard. The surgeons’ ability to determine cognitive function was validated against these data, based on their reporting of cognitive function to the NHFR.

### The Norwegian hip fracture register

The NHFR collects epidemiological data and evaluates treatment methods of hip fractures in Norway. Data is reported by the surgeons on a one-page form containing information on the patient, including cognitive status, fracture and type of operation [20]. The form is usually filled in by the surgeons immediately postoperatively. The patients’ comorbidity is classified by the American Society of Anaesthesiologists (ASA) score, normally provided to the surgeons on request by an anaesthesiologist [20]. The surgeons have the following alternatives when answering the question on chronic cognitive impairment: “Yes”, “No” or “Uncertain”. Information on cognitive function is based on preoperative assessment of the patients or on information from the medical chart. Assessment of cognitive function in the operating theatre is usually limited by verbal interactions. The large majority of patients are operated for acute hip fracture in spinal anaesthesia. If the surgeon is in doubt of the cognitive function preoperatively, use of the Clock Drawing Test is recommended [24]. As hip fracture surgery often is performed as an emergency procedure, by the surgeon on call and during evenings/weekends, the surgeon may have had limited time to study the patients’ medical chart. Further, peri-operative presence of delirium may complicate the assessment of cognitive function.

### Patient selection and case definition

**Fig. 1:**
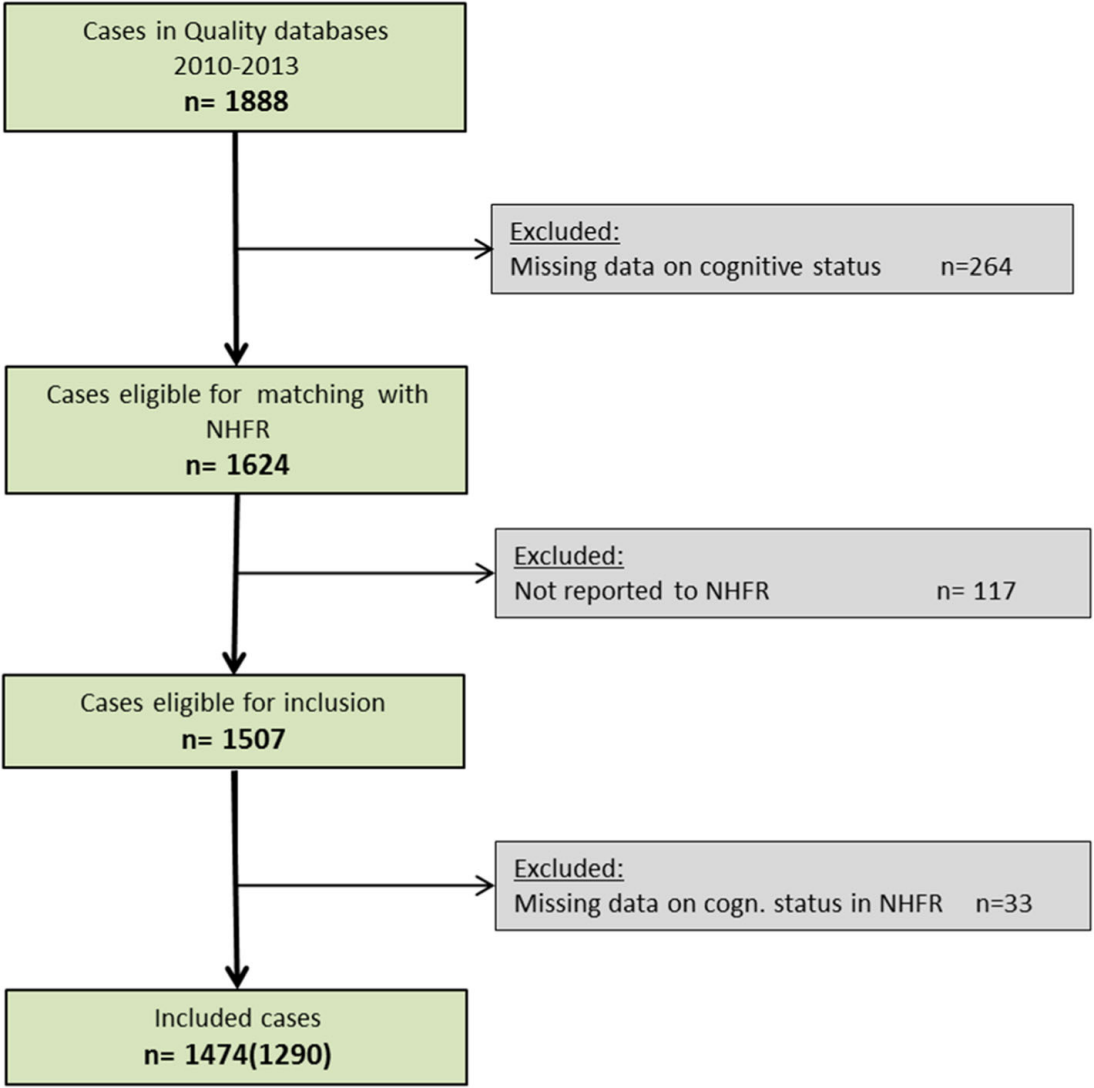
Flowchart of patient selection using dementia and/or IQCODE (using only documented dementia in parentheses)

In the period 2010–2013, 1888 primary hip fracture operations were reported to the quality databases at HDH (*n* = 242) and DH (*n* = 1646). Patients with missing data on cognitive status were excluded from further analysis (*n* = 264) (Fig. 1).

After exclusion of cases not found in the NHFR (*n* = 117) and cases with no information on cognitive status in the NHFR (*n* = 33), 1474 patients with fractures were included in the validation analyses. This included hip fracture patients with the information on dementia in the medical chart and/or IQCODE-score in the hospital quality database. Of these, 1290 patients had information on dementia from the medical chart and 507 patients had IQCODE registered in the quality databases (Fig. 1).

A cut-off point of 3.3–3.6 on IQCODE has been used for detecting dementia in community settings, while 3.44–4.0 has been used in hospital settings [23]. Accordingly, separate analyses were conducted with three different definitions of cognitive impairment in the local databases: 1) Presence of dementia documented in patient’s medical chart. 2) IQCODE > 3.44 and/or dementia. 3) IQCODE > 4 and/or dementia.

### Statistical analysis

Validation analyses were performed on the 1474 fracture patients where we had information on cognitive function in the NHFR and information on cognitive status in the local databases, either from the IQCODE score, a dementia diagnosis from medical charts, or both records. Information in the local databases was defined as a reference standard which the surgeons’ reports were validated against. Sensitivity, specificity, positive predictive value, and negative predictive value for the surgeons` reports were calculated. The patients for whom the surgeon had marked “uncertain” on chronic cognitive impairment were grouped together with patients classified with no cognitive impairment.

Pearson’s chi-square test was used for comparison of categorical variables and analysis of variance (ANOVA) was used for continuous variables. *P*-values < 0.05 were considered statistically significant. We used the statistical software packages IBM SPSS Statistics, version 23.0, for Windows and the statistical analyses.

## Results

### Baseline data and operation methods

Of the 1474 hip fracture patients included from the NHFR, 457 (31%) were classified by the surgeon as cognitively impaired and 870 (59%) as cognitively well-functioning. In 147 cases (10%), the surgeon had been uncertain of the patients´ cognitive function. The patients with chronic cognitive impairment were on average 3.6 years older and had a higher ASA score than the patients without cognitive impairment (Table 1). Most (74%) of the patients with chronic cognitive impairment were classified as ASA 3 or higher.

**Table 1:** Baseline data according to cognitive function in the Norwegian Hip Fracture Register

	Total	Cognitive impairment			*p*-value
		No	Uncertain	Yes	
Total n (%)	1.474	870 (59.0)	147 (10.0)	457 (31.0)	
Women (%)	1.111 (75.4)	651 (74.8)	100 (68.0)	360 (78.8)	0.026
Mean age (SD)	84.2 (7.9)	82.8 (8.3)	85.4 (7.2)	86.4 (6.8)	< 0.001^#^
Age group (%)					< 0.001^*^
< 75	196 (13.3)	153 (17.6)	13 (8.8)	30 (6.6)	
75–79	181 (12.3)	124 (14.3)	16 (10.9)	41 (9.0)	
80–84	265 (18.0)	161 (18.5)	25 (17.0)	79 (17.3)	
85–89	430 (29.2)	239 (27.5)	47 (32.0)	144 (31.5)	
≥ 90	402 (27.3)	193 (22.2)	46 (31.3)	163 (35.7)	
ASA class (%)					< 0.001^*^
ASA 1	26 (1.8)	26 (3.0)	0 (0)	0 (0)	
ASA 2	546 (37.0)	392 (45.1)	39 (26.5)	115 (25.2)	
ASA 3	847 (57.5)	425 (48.9)	102 (69.4)	320 (70.0)	
ASA 4	52 (3.5)	26 (3.0)	6 (4.1)	20 (4.4)	
Missing ASA	3 (0.2)	1 (0.1)	0 (0)	2 (0.4)	
Fracture type (%)					0.458
Undisplaced FNF	220 (14.9)	138 (15.9)	20 (13.6)	62 (13.6)	
Displaced FNF	606 (41.1)	352 (40.5)	62 (42.2)	192 (42.0)	
Trochanteric fracture	550 (37.3)	319 (36.7)	61 (41.5)	170 (37.2)	
Subtrochanteric	67 (4.5)	42 (4.8)	4 (2.7)	21 (4.6)	
Other^a^	31 (2.1)	19 (2,2)	0 (0)	12 (2.6)	
Primary operation (%)					0.909
Screw osteosynthesis	230 (15.6)	142 (16.3)	23 (15.6)	65 (14.2)	
Hemiarthroplasty	598 (40.6)	349 (40.1)	59 (40.1)	190 (41.6)	
Sliding hip screw	630 (42.7)	367 (42.2)	65 (44.2)	198 (43.3)	
Other^b^	16 (1.1)	12 (1.4)	0 (0)	4 (0.9)	

* = ANOVA ^**#**^ **=** Pearson’s chi square

*ASA* American society of anaesthesiologists

*FNF* Fracture of femoral neck

*AO/OTA* AO/Orthopaedic Trauma Association

Other ^a^fracture types including basocervikal fractures

Other ^b^operation methods including intramedullary nail

There were no statistically significant differences in the surgical methods used or type of fracture between the groups (Table 1).

The mean IQCODE score was 3.47 for hip fracture patients classified as not having cognitive impairment and 4.56 for hip fracture patients classified as cognitively impaired (Table 2).

**Table 2:** Baseline IQCODE

Cognitive impairment in NHFR	Numbers	Mean	Min	Max	Std.Deviation
No	340	3.47	2.87	5.00	0.567
Uncertain	58	3.98	3.00	5.00	0.652
Yes	109	4.56	3.00	5.00	0.616
Total	507	3.76	2.87	5.00	0.738

### Validation of data on cognitive function reported by orthopaedic surgeons

We used three different methods to identify chronic cognitive impairment. First, a diagnosis of dementia in the hospital chart was used as the reference for chronic cognitive impairment. In this analysis, the sensitivity of the orthopaedic surgeons` evaluation of chronic cognitive impairment reported to the NHFR was 80%. Secondly, when defining chronic cognitive impairment as a diagnosis of dementia and or an IQCODE > 4, the sensitivity was 69%. Lastly, when the reference for chronic cognitive impairment was a diagnosis of dementia or an IQCODE > 3.44, the sensitivity was 62%.

The specificity of the data in the NHFR increased from 88% using dementia diagnosis to 90% also using IQCODE (both > 4.0 and > 3.44). The positive predictive value increased from 72% using dementia diagnosis as a validation criterion to 78 and 79% including IQCODE > 4.0 and > 3.44. The negative predictive value decreased from 92% using dementia diagnosis as validation criteria to 84 and 79% using IQCODE > 4.0 and > 3.44 (Tables 3 and 4).

**Table 3:** Validation comparison of surgeons’ reporting of cognitive impairment and information on cognitive function in local databases

Local Databases	Norwegian Hip Fracture Register		
	Cognitive impairment	Uncertain	No cognitive impairment
Dementia			
Cognitive impairment (%)	279 (71.5)	23 (17.7)	49 (6.4)
No cognitive impairment (%)	111 (28.5)	107 (82.3)	721 (93.6)
Total (%)	390 (100)	130 (100)	770 (100)
Dementia and/or IQCODE > 3.44			
Cognitive impairment (%)	363 (79.4)	60 (40.8)	159 (18.3)
No cognitive impairment (%)	94 (20.6)	87 (59.2)	711 (81.7)
Total (%)	457 (100)	147 (100)	870 (100)
Dementia and/or IQCODE > 4.0			
Cognitive impairment (%)	357 (78.1)	52 (35.4)	107 (12.3)
No cognitive impairment (%)	100 (21.9)	95 (64.6)	763 (87.7)
Total (%)	457 (100)	147 (100)	870 (100)

**Table 4:** Validation of cognitive impairment reported by the surgeons using dementia and/or IQCODE

	Validation criteria		
	Dementia^a^	Dementia and/or IQCODE > 3.44^b^	Dementia and/or IQCODE > 4.0^b^
Sensitivity (CI)	79.5%	62.4%	69.2%
Specificity (CI)	88.2%	89.5%	89.6%
Positive predictive value (CI)	71.5%	79.4%	78.1%
Negative predictive value (CI)	92.0%	78.5%	84.4%

^a^Dementia registered in patients` medical journal

^b^Dementia registered in patients` medical journal and/or IQCODE> 3.44 vs. > 4.0 registered in the local hospital database

Sensitivity and negative predictive value increased with higher IQCODE cut-off and were highest when using dementia diagnosis as a reference. Specificity remained the same in all definitions. Positive predictive value decreased with increasing values for the cut-off on the IQCODE and with a previous diagnosis of dementia.

## Discussion

The orthopaedic surgeons reported chronic cognitive impairment to the NHFR in 31% of the hip fracture patients. Comparison of data on cognitive function from the hospital databases with data reported by the orthopaedic surgeons to the Norwegian Hip Fracture Register on the same patients showed high specificity and high negative predictive value. This indicates that it is easier to recognize patients without cognitive impairment among hip fracture patients and that the numbers of false positive and false negative results were low. The orthopaedic surgeons had an acceptable and clinically relevant ability to identify chronic cognitive impairment, and they did better in identifying patients with more severe cognitive impairment.

Dementia is a diagnosis with specific criteria in the ICD-10 system [25]. It is a chronic disorder characterized by an impairment of cognitive function of at least six months’ duration. A sound dementia assessment cannot be conducted during acute illness, such as during a hospitalization for a hip fracture. Delirium is an acute state of confusion which can be triggered by causes such as a fracture or an infection in vulnerable patients. Dementia can be mild or more severe and may be difficult to differentiate from delirium in an acute peri-operative setting. Our analysis does not consider the different types and different stages of cognitive impairment. Young patients in an early stage of dementia and living at home might differ from patients living in nursing homes with end stage dementia, with regard to rehabilitation potential [26]. Ranhoff et al. have reported that the rehabilitation potential in older hip fracture patients varies and that different care pathways are needed in the rehabilitation process [27]. We did not find any clinically relevant difference in surgical treatment of cognitively well-functioning and cognitively impaired patients.

### Strengths and weaknesses

The major advantage of the present study is the large number of patients. We had data from two different hospitals located in two different cities and compared the data reported from the orthopaedic surgeons with the data reported by specialized geriatric teams in the same hospitals. As both hospitals had orthogeriatric teams, the findings in the present study may, however, not be representative of results that could be achieved at other orthopaedic wards without orthogeriatric services. Surgeons at these two hospitals might be more attuned to discovering chronic cognitive impairment compared to surgeons in hospitals without orthogeriatric resources. Using data from only two hospitals increases the risk of selection bias. However, validation is dependent on correct data from established databases. We decided to use data from these two specific hospitals since both had long experience in orthogeriatric care and had developed good and complete quality databases prior to our study. An alternative method to validate the orthopaedic surgeons` ability to determine cognitive function would have been to perform a retrospective chart review. We were unable to do this due to resource constraints and we are uncertain of the extent to which the charts of hip fracture patients would contain the information necessary to evaluate cognitive function. Taking advantage of already existing quality databases with information on cognitive function enabled us to produce valid estimates of cognitive impairment, and represented a method for validating the surgeons’ ability to determine the patients’ chronic cognitive function in these hospitals.

The percentage of chronic cognitive impairment reported from the two hospitals was similar to the percentage of chronic cognitively impaired patients at all hospitals reporting to the NHFR in the observed period. Further, the baseline data for these two hospitals were similar to the baseline data found for all patients registered in the NHFR [28]. This indicates that patients in the two hospitals are representative for all Norwegian hospitals treating patients with hip fractures.

Our results on prevalence of chronic cognitive impairment are similar to epidemiological studies, showing a high number of hip fracture patients having cognitive impairment and dementia [4].

To our knowledge, no previous studies on orthopaedic surgeons’ ability to determine cognitive function in hip fracture patients have been performed. Clinicians often have a higher correlation of agreement for negative than positive diagnoses. de Vet advocates using measurement of agreement rather than Cohen’s kappa, and that there will always be more agreement in the largest group of any analysis, which in our study was the patients without cognitive impairment [29].

We analysed the data with different cut-off points of IQCODE, to show the variation in the results using different methods. Finally, we chose the results using both dementia and IQCODE > 4.0. This reflects the heterogeneity in the material and IQCODE > 4.0 is normally used in inpatient settings such as hospitals, where our patients were located.

Comparing the data on chronic cognitive impairment from the two quality databases with the information in the NHFR using three different methods (diagnosis of dementia, diagnosis of dementia and/or IQCODE > 3.44, and diagnosis of dementia and/or IQCODE > 4.0) led to somewhat different results. This demonstrates the need to know the prevalence in the population when considering positive and negative predictive value. In our population of hip fracture patients, the prevalence of chronic cognitive impairment is high and therefore gives higher positive and negative predictive values than in other populations [30].

Our results showed that surgeons identified cognitively well-functioning patients with a high negative predictive value. On the other hand, one out of five patients reported as chronic cognitively impaired to the NHFR by surgeons had no cognitive impairment according to the diagnosis in the database, and the positive predictive value of chronic cognitive impairment using dementia diagnosis and/or IQCODE > 4 as reference was 78.1%. This reflects the uncertainty in classifying patients’ chronic cognitive function in an acute setting following a hip fracture. Presence of delirium probably increases this uncertainty.

Alternative methods to detect cognitive impairment and delirium in hip fracture patients could be the Abbreviated Mental Test (AMT) and the 4 ‘A’s Test (4AT) [31–33]. AMT and 4AT can be performed by nurses after brief training [34] . These tests are recommended in the recently published Norwegian interdisciplinary guidelines on hip fracture care [35].

## Conclusion

By comparing data on chronic cognitive function reported by orthopaedic surgeons in the NHFR with data from hospital quality databases on the same patients, we found the orthopaedic surgeons’ ability to determine chronic cognitive function in hip fracture patients to be satisfactory.

Cognitively well-functioning patients were easier to identify than patients with chronic cognitive impairment. The surgical treatment of hip fractures was similar in patients with chronic cognitive impairment and cognitively well-functioning patients. The surgeons had an acceptable ability to identify and report chronic cognitive impairment in the peri-operative period, indicating that the NHFR is a valuable resource for future registry-based research on hip fracture patients, including those with chronic cognitive impairment.

## Abbreviations

4AT: 4 ‘A’s Test
AMT: Abbreviated mental test
ANOVA: Analysis of variance
ASA: American Society of Anaesthesiologists
DH: Diakonhjemmet Hospital
HDH: Haraldsplass Deaconess Hospital
IQCODE: Informant Questionnaire on cognitive decline in the elderly
NHFR: Norwegian hip fracture register

## References

[1] Stoen RO, Nordsletten L, Meyer HE, Frihagen JF, Falch JA, Lofthus CM (2012). Hip fracture incidence is decreasing in the high incidence area of Oslo, Norway. Osteoporos Int.

[2] Gjertsen JE, Fevang JM, Matre K, Vinje T, Engesaeter LB (2011). Clinical outcome after undisplaced femoral neck fractures. Acta Orthop.

[3] Seitz DP (2014). Examining the effects of dementia on postoperative outcomes of older adults with hip fractures.

[4] Seitz DP, Adunuri N, Gill SS, Rochon PA (2011). Prevalence of dementia and cognitive impairment among older adults with hip fractures. J Am Med Dir Assoc.

[5] Zhao Y, Shen L, Ji HF (2012). Alzheimer’s disease and risk of hip fracture: a meta-analysis study. ScientificWorldJournal.

[6] Tolppanen AM, Lavikainen P, Soininen H, Hartikainen S (2013). Incident hip fractures among community dwelling persons with Alzheimer's disease in a Finnish nationwide register-based cohort. PLoS One.

[7] Mundi S, Chaudhry H, Bhandari M (2014). Systematic review on the inclusion of patients with cognitive impairment in hip fracture trials: a missed opportunity?. Can J Surg.

[8] Gjertsen JE, Vinje T, Lie SA, Engesaeter LB, Havelin LI, Furnes O, Fevang JM (2008). Patient satisfaction, pain, and quality of life 4 months after displaced femoral neck fractures: a comparison of 663 fractures treated with internal fixation and 906 with bipolar hemiarthroplasty reported to the Norwegian hip fracture register. Acta Orthop.

[9] Gjertsen JE, Vinje T, Engesaeter LB, Lie SA, Havelin LI, Furnes O, Fevang JM (2010). Internal screw fixation compared with bipolar hemiarthroplasty for treatment of displaced femoral neck fractures in elderly patients. J Bone Joint Surg Am.

[10] Mukka S, Knutsson B, Krupic F, Sayed-Noor AS (2017). The influence of cognitive status on outcome and walking ability after hemiarthroplasty for femoral neck fracture: a prospective cohort study. Eur J Orthop Surg Traumatol.

[11] Corrada MM, Brookmeyer R, Paganini-Hill A, Berlau D, Kawas CH (2010). Dementia incidence continues to increase with age in the oldest old: the 90+ study. Ann Neurol.

[12] Juliebo V, Bjoro K, Krogseth M, Skovlund E, Ranhoff AH, Wyller TB (2009). Risk factors for preoperative and postoperative delirium in elderly patients with hip fracture. J Am Geriatr Soc.

[13] Krogseth M, Watne LO, Juliebo V, Skovlund E, Engedal K, Frihagen F, Wyller TB (2016). Delirium is a risk factor for further cognitive decline in cognitively impaired hip fracture patients. Arch Gerontol Geriatr.

[14] Bitch M, Foss N, Kristensen B, Kehlet H (2004). Patogenesis of and management strategies for postoperative delirium after hip fracture: a review. Acta Orthop Scand.

[15] Lundstrom M, Edlund A, Bucht G, Karlsson S, Gustafson Y (2003). Dementia after delirium in patients with femoral neck fractures. J Am Geriatr Soc.

[16] Krogseth M, Wyller TB, Engedal K, Juliebo V (2011). Delirium is an important predictor of incident dementia among elderly hip fracture patients. Dement Geriatr Cogn Disord.

[17] Mosk CA, Mus M, Vroemen JP, van der Ploeg T, Vos DI, Elmans LH, van der Laan L (2017). Dementia and delirium, the outcomes in elderly hip fracture patients. Clin Interv Aging.

[18] Dubljanin-Racpopoc E, Matanovic D, Bumbasirevic M (2010). The impact of cognitive impairment at admission on short-term functional outcome of elderly hip fracture patients. Srp Arh Celok Lek.

[19] Zerah L, Cohen-Bittan J, Raux M, Meziere A, Tourette C, Neri C, Verny M, Riou B, Khiami F, Boddaert J (2017). Association between cognitive status before surgery and outcomes in elderly patients with hip fracture in a dedicated Orthogeriatric care pathway. J Alzheimers Dis.

[20] Gjertsen JE, Engesaeter LB, Furnes O, Havelin LI, Steindal K, Vinje T, Fevang JM (2008). The Norwegian hip fracture register: experiences after the first 2 years and 15,576 reported operations. Acta Orthop.

[21] Harrison JK, Fearon P, Noel-Storr AH, McShane R, Stott DJ, Quinn TJ. Informant Questionnaire on Cognitive Decline in the Elderly (IQCODE) for the diagnosis of dementia within a secondary care setting. Cochrane Database Syst Rev. 2015:Cd010772. 10.1002/14651858.CD010772.pub2 25754745

[22] Quinn TJ, Fearon P, Noel-Storr AH, Young C, McShane R, Stott DJ. Informant questionnaire on cognitive decline in the elderly (IQCODE) for the diagnosis of dementia within community dwelling populations. Cochrane Database Syst Rev. 2014;(4):CD010079. 10.1002/14651858.CD010079.pub2 24719028

[23] Jorm AF (2004). The informant questionnaire on cognitive decline in the elderly (IQCODE): a review. Int Psychogeriatr.

[24] Amodeo S, Mainland BJ, Herrmann N, Shulman KI (2015). The times they are a-Changin': clock drawing and prediction of dementia. J Geriatr Psychiatry Neurol.

[25] Naik M, Nygaard HA (2008). Diagnosing dementia -- ICD-10 not so bad after all: a comparison between dementia criteria according to DSM-IV and ICD-10. Int J Geriatr Psychiatry.

[26] Prestmo A, Saltvedt I, Helbostad JL, Taraldsen K, Thingstad P, Lydersen S, Sletvold O (2016). Who benefits from orthogeriatric treatment? Results from the Trondheim hip-fracture trial. BMC Geriatr.

[27] Ranhoff AH, Holvik K, Martinsen MI, Domaas K, Solheim LF (2010). Older hip fracture patients: three groups with different needs. BMC Geriatr.

[28] Gjertsen JE, Fenstad AM, Leonardsson O, Engesaeter LB, Karrholm J, Furnes O, Garellick G, Rogmark C (2014). Hemiarthroplasties after hip fractures in Norway and Sweden: a collaboration between the Norwegian and Swedish national registries. Hip Int.

[29] de Vet H. C. W., Mokkink L. B., Terwee C. B., Hoekstra O. S., Knol D. L. (2013). Clinicians are right not to like Cohen's. BMJ.

[30] Lydersen S. What is the probability of a correct result of a diagnostic test? Tidsskr Nor Laegeforen. 2017;137. 10.4045/tidsskr.17.0409 28972344

[31] Swain DG, Nightingale PG (1997). Evaluation of a shortened version of the abbreviated mental test in a series of elderly patients. Clin Rehabil.

[32] Schofield I, Stott DJ, Tolson D, McFadyen A, Monaghan J, Nelson D (2010). Screening for cognitive impairment in older people attending accident and emergency using the 4-item abbreviated mental test. Eur J Emerg Med.

[33] Bellelli G, Morandi A, Davis DH, Mazzola P, Turco R, Gentile S, Ryan T, Cash H, Guerini F, Torpilliesi T (2014). Validation of the 4AT, a new instrument for rapid delirium screening: a study in 234 hospitalised older people. Age Ageing.

[34] Mossello E, Tesi F, Di Santo SG, Mazzone A, Torrini M, Cherubini A, Bo M, Musicco M, Bianchetti A, Ferrari A (2018). Recognition of delirium features in clinical practice: data from the "delirium day 2015" National Survey. J Am Geriatr Soc.

[35] Norwegian Ortho**-**Geriatric guidelines on hip fracture treatment [http://legeforeningen.no/PageFiles/329853/Norske%20retningslinjer%20for%20tverrfaglig%20behandling%20av%20hoftebrudd.pdf].

